# Measuring the Pharmacodynamic Effects of a Novel Hsp90 Inhibitor on HER2/*neu* Expression in Mice Using ^89^Zr-DFO-Trastuzumab

**DOI:** 10.1371/journal.pone.0008859

**Published:** 2010-01-25

**Authors:** Jason P. Holland, Eloisi Caldas-Lopes, Vadim Divilov, Valerie A. Longo, Tony Taldone, Danuta Zatorska, Gabriela Chiosis, Jason S. Lewis

**Affiliations:** 1 Radiochemistry Service, Department of Radiology, Memorial Sloan-Kettering Cancer Center, Sloan-Kettering Cancer Center, New York, New York, United States of America; 2 Program in Molecular Pharmacology and Chemistry, Sloan Kettering Institute, Memorial Sloan-Kettering Cancer Center, New York, New York, United States of America; 3 Small-Animal Imaging Core Facility, Memorial Sloan-Kettering Cancer Center, New York, New York, United States of America; Chiba University Center for Forensic Mental Health, Japan

## Abstract

**Background:**

The positron-emitting radionuclide ^89^Zr (*t*_1/2_ = 3.17 days) was used to prepare ^89^Zr-radiolabeled trastuzumab for use as a radiotracer for characterizing HER2/*neu*-positive breast tumors. In addition, pharmacodynamic studies on HER2/*neu* expression levels in response to therapeutic doses of PU-H71 (a specific inhibitor of heat-shock protein 90 [Hsp90]) were conducted.

**Methodology/Principal Findings:**

Trastuzumab was functionalized with desferrioxamine B (DFO) and radiolabeled with [^89^Zr]Zr-oxalate at room temperature using modified literature methods. ImmunoPET and biodistribution experiments in female, athymic *nu*/*nu* mice bearing sub-cutaneous BT-474 (HER2/*neu* positive) and/or MDA-MB-468 (HER2/*neu* negative) tumor xenografts were conducted. The change in ^89^Zr-DFO-trastuzumab tissue uptake in response to high- and low-specific-activity formulations and co-administration of PU-H71 was evaluated by biodistribution studies, Western blot analysis and immunoPET. ^89^Zr-DFO-trastuzumab radiolabeling proceeded in high radiochemical yield and specific-activity 104.3±2.1 MBq/mg (2.82±0.05 mCi/mg of mAb). *In vitro* assays demonstrated >99% radiochemical purity with an immunoreactive fraction of 0.87±0.07. *In vivo* biodistribution experiments revealed high specific BT-474 uptake after 24, 48 and 72 h (64.68±13.06%ID/g; 71.71±10.35%ID/g and 85.18±11.10%ID/g, respectively) with retention of activity for over 120 h. Pre-treatment with PU-H71 was followed by biodistribution studies and immunoPET of ^89^Zr-DFO-trastuzumab. Expression levels of HER2/*neu* were modulated during the first 24 and 48 h post-administration (29.75±4.43%ID/g and 41.42±3.64%ID/g, respectively). By 72 h radiotracer uptake (73.64±12.17%ID/g) and Western blot analysis demonstrated that HER2/*neu* expression recovered to baseline levels.

**Conclusions/Significance:**

The results indicate that ^89^Zr-DFO-trastuzumab provides quantitative and highly-specific delineation of HER2/*neu* positive tumors, and has potential to be used to measure the efficacy of long-term treatment with Hsp90 inhibitors, like PU-H71, which display extended pharmacodynamic profiles.

## Introduction

In the era of molecular medicine, antibody-based agents offer unparalleled potential as platforms for the development of target-specific therapies.[1] Immunoconjugates are monoclonal antibodies (mAbs) or antibody fragments functionalized with cytotoxic and/or diagnostic payloads. Increasing availability of longer-lived positron-emitting radionuclides such as ^64^Cu, ^86^Y, ^89^Zr and ^124^I, and advances in chelation chemistry, have renewed interest in the use of positron emission tomography with radioimmunoconjugates (immunoPET) as a tool for providing real-time, quantitative information on physiological response to treatment.[2]–[5]

Proteins associated with the human epidermal growth-factor receptor kinase (ERBB or HER) signaling network have proved to be valuable targets for diagnostic imaging with radioimmunoconjugates due to their overexpression in various cancers phenotypes. In particular, overexpression of HER2/*neu* (also known as ERBB2) has been found to correlate with increased tumor aggression, metastatic potential, and poor prognosis for disease-free survival in patients with breast, colorectal, ovarian, lung, prostate and salivary gland tumors.[6], [7] The ERBB signaling network and the role of HER2/*neu* in cancer biology has been the subject of several excellent reviews.[7]–[11]

HER2/*neu* has emerged as a key target for anticancer drugs due to its intrinsic involvement in the phosphatidylinositol-3-kinase-Akt/protein kinase B (PI3K-Akt) and the mitogen-activated protein kinase (MAPK) pathways, both of which suppress apoptosis and promote tumor cell survival, gene transcription, angiogenesis, cellular proliferation, migration, mitosis, and differentiation.[7] Three important classes of anti-HER2/*neu* therapeutics include: mAbs directed against extracellular ligand-binding and dimerization epitopes, tyrosine-kinase (TK) inhibitors and Hsp90 inhibitors. Examples of each class include pertuzumab and trastuzumab (which block dimerization and suppress signaling by binding to extracellular domains II and IV, respectively); the HER2/*neu* TK-inhibitor lapatinib; and Hsp90 inhibitors including geldanamycin derivatives, SNX-5422, NVP-AUY922, BIIB021 and PU-H71.[7], [11]–[18]

Trastuzumab (Herceptin™, Genentech) has been exploited as both a therapeutic agent and radiotracer. Trastuzumab and related mAb-fragments have been radiolabeled with a wide range of radionuclides and quantitative immunoPET imaging has been used to monitor the effect of administering Hsp90 inhibitors on HER2/*neu* expression levels.[19]–[30] Quantification of changes in HER2/*neu* expression in response to Hsp90 treatment has the potential to facilitate patient-specific dose regimes. For example, studies using ^64^Cu-labeled trastuzumab and ^68^Ga-labeled F(ab')_2_-trastuzumab have been reported.[19]–[21] However, both the ^64^Cu- and ^68^Ga-labeled agents are sub-optimal radiotracers. The comparatively short half-life of ^64^Cu (*t*_1/2_ = 12.7 h) means that the radionuclide has decayed before the intact antibody achieves optimal biodistribution (ca. 4–5 days), and the small size the F(ab')_2_ fragments gives poor tumor-to-background tissue contrast ratios.[1], [2] In efforts to overcome these limitations in radiotracer design, Orlova *et al*.[28] and Kramer-Marek *et al*.[29] explored the potential of ^124^I- and ^18^F-labeled trastuzumab affibodies for measuring HER2/*neu* expression, respectively.

Over recent years, ^89^Zr has emerged as a promising radionuclide for use in immunoPET. In particular, facile methods for radiolabeling intact mAbs with ^89^Zr have been developed from the pioneering work of researchers at the Vrije University Medical Center (Amsterdam, The Netherlands).[30]–[46] Zirconium-89 has a number of distinct advantages which make it ideal for immunoPET: (i) the half-life of 78.41 h matches closely the extend times required for optimum biodistribution of intact mAbs, (ii) the positron yield of 22.7% is comparable to that of ^64^Cu, ^86^Y and ^124^I which improves counting statistics in PET imaging, (iii) zirconium and its ions are generally inert to biological systems and have no known biological role or function, (iv) cyclotron production of ^89^Zr *via* the (*p*,*n*) transmutation reaction using a 100% naturally abundant ^89^Y solid target is highly efficient and cost effective,[47] and (v) high purity and high specific-activity ^89^Zr is now available in various chemical forms which are suitable for radiolabeling mAbs. Despite initial concerns over the radiation dosimetry with ^89^Zr which stem from the presence of higher energy γ-rays (*E*γ = 909.2 keV, *I*γ = 99.0%), several clinical studies in Europe have shown that a variety of ^89^Zr-labeled mAbs targeting different tumor types are safe for use in humans.[44]–[46] Furthermore, these clinical studies demonstrate the benefits of ^89^Zr-labeled mAbs, which in comparison to equivalent mAbs and antibody fragments labeled with other PET radionuclides including ^64^Cu, ^68^Ga, ^86^Y and ^124^I, display higher tumor uptake, lower background tissue accumulation, improved image contrast and more facile delineation of tumors *in vivo*. The use of intact antibodies as the basis for radiotracer development, such as trastuzumab which is used routinely in the clinic worldwide, also has the advantage of expediting preclinical and clinical translation.

The work presented here describes the production of ^89^Zr-radiolabeled trastuzumab and its ability to identify HER2/*neu* positive tumors *in vivo*. During the course of our investigations, Dijkers *et al*. reported initial studies on ^89^Zr-labeled trastuzumab in the SKOV-3 xenograft model.[30] Our studies further investigate the efficacy of ^89^Zr-DFO-trastuzumab as a radiotracer for immunoPET and include detailed studies using the BT-474 (HER2/*neu* positive) and MDA-MB-468 (HER2/*neu* negative) xenograft models. Our work also complements and extends the methods of Smith-Jones *et al*.[20] which used ^68^Ga-labeled trastuzumab fragments and 17-AAG for measuring pharmacodynamic changes in HER2/*neu* expression *in vivo* to the full antibody with the novel and more potent purine-based Hsp90 inhibitor, PU-H71.[48] Studies include *in vitro* and *in vivo* by Western blot analysis, acute biodistribution studies and immunoPET. The results demonstrate ^89^Zr-radiolabeled trastuzumab has the potential to be used in the clinic as a radiotracer for both localizing and staging of HER2/*neu* positive tumors, and in the long-term measurements of the efficacy of treatment with Hsp90 inhibitors such as PU-H71 and other drugs with extended pharmacodynamic profiles.

## Materials and Methods

All chemicals, unless otherwise stated, were purchased from SigmaAldrich (St. Louis, MO) and were used as received. Water (>18.2 MΩ.cm at 25°C, Milli-Q, Millipore, Billerica, MA) was purified by passing through a 10 cm column of chelex resin (Bio-Rad Laboratories, Hercules, CA) at a flow rate <1.0 mL/min. All instruments were calibrated and maintained in accordance with previously reported routine quality-control procedures.[49] Activity measurements were made by using a Capintec CRC-15R Dose Calibrator (Capintec, Ramsey, NJ) with a calibration factor of 465 for ^89^Zr. For accurate quantification of activities, experimental samples were counted for 1 min. on a calibrated Perkin Elmer (Waltham, MA) Automatic Wizard^2^ Gamma Counter by using an energy window of 800–1000 keV for ^89^Zr (909 keV emission). ^89^Zr-radiolabeling reactions were monitored by using silica-gel impregnated glass-fibre instant thin-layer chromatography (ITLC-SG) paper (Pall Corp., East Hills, NY) and analyzed on a radio-TLC plate reader (Bioscan System 200 Imaging Scanner coupled to a Bioscan Autochanger 1000 (Bioscan Inc., Washington, DC, using Win-Scan Radio-TLC software version 2.2). Solvent systems included diethylene triamine pentaacetic acid in water (DTPA, 50 mM, pH 7) and phosphate buffered saline (PBS). Human breast cancer cell lines BT-474 and MDA-MB-468 were obtained from the American Type Culture Collection (ATCC, Manassas, VA) and were grown by serial passage. Full details for all methods are provided in the supporting information (Text S1).

### Trastuzumab Conjugation and Radiolabeling

Trastuzumab (Herceptin™, Genentech, South San Francisco, CA) was conjugated to desferrioxamine B (DFO, Calbiochem, Spring Valley, CA) by using procedures modified from those described by Verel *et al*.[36] Full details are provided in the supporting information (Text S1). Briefly, DFO-conjugated trastuzumab (DFO-trastuzumab) was prepared *via* a 6-step procedure involving: the reaction of DFO mesylate with succinic anhydride to give *N*-succinyldesferrioxamine B; protection of the *tris*-hydroxamate functional groups *via* Fe^3+^ complexation; formation of the tetrafluorophenol (TFP) activated ester; mAb conjugation; removal of the Fe^3+^ *via* transchelation to EDTA; and purification of DFO-trastuzumab by using size-exclusion chromatography and/or spin-column centrifugal separation.

Zirconium-89 was produced *via* the ^89^Y(*p*,*n*)^89^Zr transmutation reaction on an EBCO TR19/9 variable-beam energy cyclotron (Ebco Industries Inc., Richmond, British Columbia, Canada) in accordance with previously reported methods.[36], [47], [50] The [^89^Zr]Zr-oxalate was isolated in high radionuclidic and radiochemical purity (RCP) >99.99%, with a specific-activity of 195–497 MBq/µg, (5.28–13.43 mCi/µg).[47]

^89^Zr-DFO-trastuzumab was prepared by the complexation of [^89^Zr]Zr-oxalate with DFO-trastuzumab. Typical radiolabeling reactions were conducted in accordance with the following procedure. Briefly, [^89^Zr]Zr-oxalate (29.7 MBq, [805 µCi]) in 1.0 M oxalic acid (40 µL) was adjusted to pH 7.7–8.5 with 1.0 M Na_2_CO_3_(aq.). CAUTION: Acid neutralization releases CO_2_(g) and care should be taken to ensure that no radioactivity escapes the microcentrifuge vial. After CO_2_(g) evolution ceased, DFO-trastuzumab (50 µL, 5.0 mg/mL [0.25 mg of mAb], in 0.9% sterile saline) was added and the reaction was mixed gently. The reaction was incubated at room temperature for 1–2 h and complexation progress was monitored by ITLC (DTPA, 50 mM, pH 7). After 1 h, high radiolabeling yields were obtained with RCP >78(±4)%. ^89^Zr-DFO-trastuzumab was purified by using either size-exclusion chromatography (Sephadex G-25 M, PD-10 column, >30 kDa, GE Healthcare; dead-volume = 2.5 mL, eluted with 200 µL fractions of 0.9% sterile saline, Figure S1) or spin-column centrifugation (4 mL volume, >30 kDa, Amicon Ultra-4, Millipore, Billerica, MA; washed with 4×3 mL, 0.9% sterile saline). The RCP of the final ^89^Zr-DFO-trastuzumab (>70% radiochemical yield; formulation: pH 5.5–6.0; <500 µL; 0.9% sterile saline) was measured by both radio-ITLC and analytical size-exclusion chromatography (<0.74 MBq [20 µCi], ca. 5–10 µL aliquots) and was found to be >99% in all preparations. In the ITLC experiment ^89^Zr-DFO-trastuzumab and [^89^Zr]Zr-DFO remain at the baseline (*R_f_* = 0.0), whereas ^89^Zr^4+^(aq.) ions and [^89^Zr]Zr-DTPA elute with the solvent front (*R_f_* = 1.0).

### Chelate Number

The number of accessible DFO chelates conjugated to trastuzumab was measured by radiometric isotopic dilution assays following a method similar to that described by Anderson *et al* (see supporting information).[51], [52] Full details are provided in the supporting information (Text S1).

### Immunoreactivity

The immunoreactive fraction of ^89^Zr-DFO-trastuzumab samples was determined by using specific radioactive cellular-binding assays following modified procedures derived from Lindmo *et al*.[53] Briefly, BT-474 or MDA-MB-468 cells were suspended in micro-centrifuge tubes at concentrations of 5.0, 4.0, 3.0, 2.5, 2.0, 1.5, and 0.5×10^6^ cells/mL in 500 µL PBS (pH 7.4). Aliquots of ^89^Zr-DFO-trastuzumab (50 µL of a stock solution of <0.37 kBq, [<0.01 mCi] in 10 mL of 1% bovine serum albumin [BSA]; 2 kBq [0.05 µCi], 0.02 µg of mAb) were added to each tube (*n* = 3; final volume: 550 µL) and the samples incubated on an orbital mixer for 60 min. at room temperature. Cells were then pelleted by centrifugation (600G for 2 min.), resuspended and washed twice with ice-cold PBS before removing the supernatant and counting the ^89^Zr-activity associated with the cell pellet. The count data were background corrected and compared with the total number of counts in control samples. Competitive inhibition (blocking) studies were conducted by using the same procedure but with the addition of non-radiolabeled trastuzumab (50 µL, 2 mg/mL in 1% BSA, [>5000-fold excess mAb; 100 µg]) to the ^89^Zr-DFO-trastuzumab solutions. Immunoreactive fractions were determined by linear regression analysis of a plot of (total/bound) activity *versus* (1/[normalized cell concentration]), and calculated as 1/*y*-intercept. Full details are provided in the supporting information (Text S1).

### Stability Studies

The stability of ^89^Zr-DFO-trastuzumab with respect to radiochemical purity, loss of radioactivity from the mAb and change in immunoreactivity was investigated *in vitro* by incubation in solutions of 0.9% saline, DTPA (50 mM, pH 7) and human serum for 7 days at 37°C. The radiochemical purity and degree of transchelation to DTPA was determined by radio-ITLC and γ-counting, and the immunoreactive fraction was measured by the cellular-binding assay (*vide supra*).

### Xenograft Models

All animal experiments were conducted in compliance with the guidelines of the Institutional Animal Care and Use Committee (IACUC) of MSKCC. Female athymic *nu*/*nu* mice (NCRNU-M, 20–22 g, 6–8 weeks old) were obtained from Taconic Farms Inc. (Hudson, NY), and were acclimated to the MSKCC vivarium for 1 week prior to implanting tumors. Mice were provided with food and water *ad libitum*. Tumors were induced on a shoulder by sub-cutaneous (s.c.) injection of 5.0–10.0 (×10^6^) cells in a 200 µL cell suspension of a 1:1v/v mixture of media with reconstituted basement membrane (BD Matrigel™, Collaborative Biomedical Products Inc., Bedford, MA).[54] For animals bearing two tumors used in the immunoPET studies, BT-474 and MDA-MB-468 cells were implanted s.c. on the left and right shoulder/flank, respectively. Palpable tumors developed after a period of 14–18 days and the tumor volume (*V*/mm^3^) was estimated by external vernier caliper measurements (Full details are provided in the supporting information (Text S1)).[55]

### Acute Biodistribution Studies

Acute *in vivo* biodistribution studies were conducted to evaluate the uptake of ^89^Zr-DFO-trastuzumab in mice bearing s.c. BT-474 tumors (90–170 mm^3^). Mice were randomized before the study and were warmed gently with a heat lamp 5 min. before administering ^89^Zr-DFO-trastuzumab (0.55–0.74 MBq, [15–20 µCi], 5–7 µg of mAb, in 200 µL 0.9% sterile saline) *via* intravenous (i.v.) tail-vein injection (*t* = 0 h). Animals (*n* = 3–8, per group) were euthanized by CO_2_(g) asphyxiation at 1, 12, 24, 48, 72 and 120 h post-injection and 12 organs (including the tumor) were removed, rinsed in water, dried in air for 5 min., weighed and counted in a gamma-counter for ^89^Zr-activity. The mass of ^89^Zr-DFO-trastuzumab formulation injected into each animal was measured and used to determine the total number of counts (counts per minute, [c.p.m.]) by comparison to a standard syringe containing and independently measured activity and mass. Count data were background and decay-corrected to the time of injection and the percent injected dose per gram (%ID/g) for each tissue sample calculated by normalization to the total activity injected.

The effects of pre-administering PU-H71 (intraperitoneal (i.p.), 75 mg/kg, 200 µL of PU-H71-hydrochloride dissolved in 10 mM phosphate buffer [PB]) 4 h prior to i.v. injection of ^89^Zr-DFO-trastuzumab, were also investigated by acute biodistribution studies at 12, 24, 48 and 72 h.

Competitive inhibition studies were also performed to investigate the specificity of ^89^Zr-DFO-trastuzumab for HER2/*neu*. Non-radiolabeled trastuzumab (10 mg/kg solution in 0.9% sterile saline, 0.2 mg/mouse) was added to the ^89^Zr-DFO-trastuzumab formulation to reduce the specific-activity (30–40-fold decrease: 2.60–3.65 MBq/mg [0.07–0.1 mCi/mg]) and biodistribution studies were performed at 24 h post-i.v. administration.

### Pharmacodynamic Studies

Changes in the expression levels of HER2/*neu* in response to Hsp90 inhibition were investigated by *in vivo* pharmacodynamic (PD) studies. Prior to BT-474 cell inoculation, mice were implanted s.c. with 0.72 mg sustained release 17β-estradiol pellets by using a 10 g trocar. Tumors were established as described above and were allowed to grow to 6–8 mm in diameter (120–270 mm^3^) before commencing treatment. Mice (*n* = 2, per group) bearing s.c. BT-474 tumors were administered PU-H71 (as the hydrochloride salt, i.p., 75 mg/kg, 200 µL, 10 mM PB). Animals were euthanized by CO_2_(g) asphyxiation at 12, 24, 48, 72 and 96 h post-i.p. administration of PU-H71 and tumors were collected. For protein analysis, tumors were homogenized in SDS lysis buffer (50 mM Tris, pH 7.4, 150 mM NaCl, and 1% Nonidet P-40) and subjected to Western blot analysis (*vide infra*).

### Western Blots

BT-474 cells were grown to 60–70% confluence and treated with either PU-H71 (in 10 mM PB) at various concentrations or DMSO for the specified time. Lysates were prepared by using 50 mM Tris, pH 7.4, 150 mM NaCl, and 1% Nonidet P-40 lysis buffer. Protein concentrations were determined by using the bicinchoninic acid (BCA) Protein Assay kit (Pierce, Rockford, IL) in accordance with the manufacturer's protocol. Protein lysates (20–100 µg) were resolved electrophoretically by SDS-PAGE, transferred to nitrocellulose membrane, and then probed with the specified primary antibodies: anti-HER2 from rabbit (1∶250, 280004; Zymed Laboratories Inc., South San Francisco, CA); anti-PARP (p85 fragment) from rabbit (1∶250, G7341; Promega, Madison, WI); anti-phospho-Akt (Ser 473) from rabbit (1∶500, 9271S; Cell Signaling); anti-Akt from rabbit (1∶500, 9272; Cell Signaling Technology Inc., Danvers, MA); anti-Hsp70 from mouse (1∶500, SPA-810; Stressgen, Ann Arbor, MI); anti-Hsp90 from mouse (1∶500, SPA-830; Stressgen); and anti-Raf-1 from rabbit (1∶300, sc-133; Santa Cruz Biotechnology Inc., Santa Cruz, CA). Membranes were then incubated with a 1∶5,000 dilution of a corresponding peroxidase-conjugated secondary antibody. Equal loading of the protein samples was confirmed by parallel Western blots for β-actin (1∶5,000, ab8227–50; Abcam Inc., Cambridge, MA). Detection was performed by using the ECL-Enhanced Chemiluminescence Detection System (Amersham Biosciences, Fairfield, CT) according to the manufacturer's instructions. Blots were visualized by autoradiography.[48] Gels were scanned using Adobe Photoshop 7.0.1 and semi-quantitative densitometric analysis was performed by using Un-Scan-It software.

### Small-Animal ImmunoPET Imaging

PET imaging experiments were conducted on a microPET Focus 120 rodent scanner (Concorde Microsystems).[56] Mice were administered ^89^Zr-DFO-trastumab formulations (8.50–9.25 MBq, [230–250 µCi], 80–90 µg of mAb, in 200 µL 0.9% sterile saline for injection) *via* tail-vein injection. The effects of pre-administering PU-H71 (i.p., 75 mg/kg, 200 µL of PU-H71-hydrochloride dissolved in 10 mM phosphate buffer [PB]) or vehicle-control, 4 h prior to i.v. injection of ^89^Zr-DFO-trastuzumab, were also investigated by immunoPET. Approximately 5 minutes prior to recording PET images, mice were anesthetized by inhalation of 2% isoflurane (Baxter Healthcare, Deerfield, IL)/oxygen gas mixture and placed on the scanner bed; anesthesia was maintained using 1% isoflurane/oxygen gas mixture. PET data were recorded at various time-points between 1–120 h. List-mode data were acquired for 10–30 min. using a γ-ray energy window of 350–750 keV, and a coincidence timing window of 6 ns. Images were analyzed by using ASIPro VM™ software (Concorde Microsystems). Full experimental details are presented in the supporting information. Full details are provided in the supporting information (Text S1).

### Statistical Analysis

Data were analyzed by the unpaired, two-tailed Student's *t*-test. Differences at the 95% confidence level (*P*<0.05) were considered to be statistically significant.

## Results and Discussion

During the course of our investigations, Dijkers *et al*. reported initial studies using ^89^Zr-labeled trastuzumab in SKOV-3 (HER2/*neu* positive) xenograft models.[30] In these studies we have extended the use ^89^Zr-DFO-trastuzumab to investigate uptake in the BT-474 (HER2/*neu* positive) and MDA-MB-468 (HER2/*neu* negative) xenograft models. We have also explored the potential of ^89^Zr-DFO-trastuzumab to be used as a non-invasive radiotracer for qualitative and quantitative delineation of the efficacy of Hsp90 inhibitor treatment (namely PU-H71) on HER2/*neu* expression levels *in vivo*.

### ^89^Zr-DFO-Trastuzumab Was Produced in High Yield and Specific-Activity

Trastuzumab was functionalized with the hexadentate, trihydroxamate chelate, desferrioxamine B (DFO) by using bioconjugation methods modified from the pioneering work of Verel *et al*.[36] Previous studies have shown that although DTPA can be used for chelation and radiolabeling of mAbs with ^89^Zr^4+^ ions, demetalation occurs *in vivo* and at present DFO remains the chelate of choice.[31], [33], [47] Despite the multi-step functionalization methods involving protection/deprotection using Fe^3+^ ions the conjugation and purification chemistry was found to proceed in moderate-to-high yield (60–78%) with high purity. Radiolabeling of sterile DFO-trastuzumab with either [^89^Zr]Zr-oxalate or [^89^Zr]Zr-chloride was achieved at room temperature in alkaline solutions (pH 7.7–8.5) with crude radiochemical yields (>95%, *n* = 15). Facile purification of ^89^Zr-DFO-trastuzumab from small-molecule radiolabeled impurities can be achieved by using either size-exclusion chromatography or spin-column centrifugation. The final radiochemical yield of purified ^89^Zr-DFO-trastuzumab was >70% and the product was formulated in either PBS or 0.9% sterile saline with RCP >99% (*n* = 12) and a specific-activity of 104.3±2.1 MBq/mg (2.82±0.05 mCi/mg) of mAb. The specific-activity obtained in our studies compares favorably with the previously reported specific-activity of 67.2±2.4 MBq/mg (1.82±0.07 mCi/mg).[30] Isotopic dilution assays revealed an average of 6.8±0.8 accessible chelates per mAb.

### ^89^Zr-DFO-Trastuzumab Is Stable and Immunoreactive *In Vitro*

Incubation of ^89^Zr-DFO-trastuzumab in either 0.9% sterile saline or human serum for 7 days at 37°C revealed <2% decrease in RCP (*via* demetalation) with a ∼27% decrease in immunoreactive fraction (0.60±0.02). In contrast, experiments conducted in DTPA (50 mM, pH 7) showed a 55±2% decrease in RCP after 7 days. DTPA represents a particularly strong ligand challenge and overall these results are consistent with other reports which indicate that ^89^Zr-DFO-mAbs display high kinetic stability and are suitable for further *in vivo* studies.[30], [35], [40], [43]–[45]

**Figure 1:**
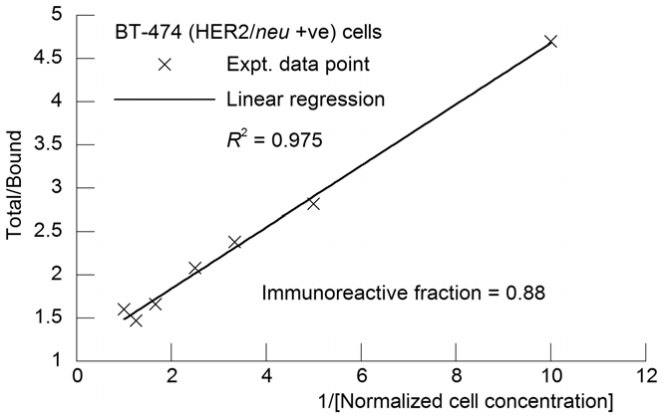
Plot of the (total/bound) activity *versus* (1/[normalized cell concentration]), used to calculate the immunoreactivity fraction of ^89^Zr-DFO-trastuzumab in BT-474 (HER2/*neu* positive) cells by extrapolation to infinite antigen excess (1/*y*-intercept).

The immunoreactive fraction of the ^89^Zr-DFO-trastuzumab formulations was measured by specific *in vitro* cellular association assays using BT-474, HER2/*neu* positive cells prior to each *in vivo* experiment (Figure 1).[53] The average immunoreactive fraction of ^89^Zr-DFO-trastuzumab was found to be 0.87±0.07 (*n* = 18). Control experiments (*n* = 3) using the HER2/*neu* negative MDA-MB-468 cell line or BT-474 cells with low specific-activity formulations (0.37±0.1 MBq/mg [10.2±0.2 µCi/mg]) in which a >5000-fold excess of non-radiolabeled trastuzumab was added to the reactions showed no binding and demonstrated the specificity of ^89^Zr-DFO-trastuzumab for HER2/*neu* expressing cells (Figures S2A and S2B).

### In Vitro Pharmacodynamic Studies Demonstrate the Effects of PU-H71 on HER2/neu Expression

**Figure 2:**
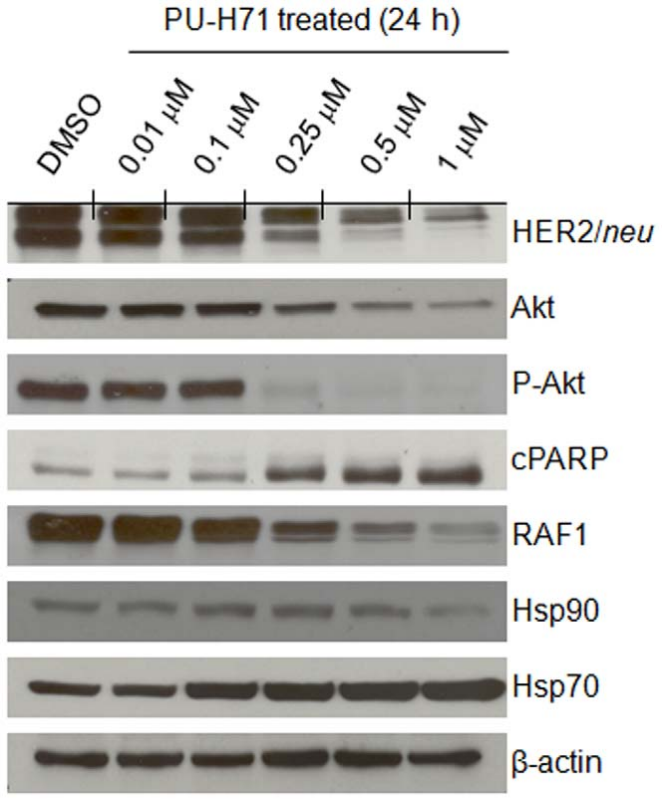
*In vitro* Western blots showing the change in protein expression levels in response to treatment with PU-H71 at 0.01–1 µM concentrations. DMSO vehicle treated experiments and β-actin are include as controls. Proteins investigated include: HER2/*neu*; Akt; phosphorylated Akt (P-Akt); the apoptosis marker, cleaved poly (ADP-ribose) polymerase (cPARP); RAF1; Hsp90; Hsp70; and β-actin as a protein loading control.

In HER2/*neu* positive tumors, Hsp90 has been shown to regulate the expression levels of a variety of onco-proteins including HER2/*neu*.[18], [57] *In vitro* Western blot studies showed that treatment of BT-474 cells with varying concentrations of PU-H71 (1–0.01 µM, Figure S3) for 24 h led to changes in expression levels of proteins associated with the ERBB signaling cascade (Figure 2). In each case, expression levels of the Hsp90-independent protein β-actin, was used as a protein loading control. Consistent with the Hsp90-mediated mechanism of action, PU-H71 treatment at concentrations ≥0.25 µM, decreased expression levels of Hsp90 client onco-proteins including HER2/*neu*, Akt and RAF1, compared to DMSO-treated controls. Degradation of Akt and phosphorylated Akt (P-Akt) leads to inactivation of cell survival pathways and the observed concomitant increase in levels of cleaved poly-(ADP-ribose) polymerase (cPARP) is indicative of apoptosis. Decreased expression levels of RAF1 demonstrate that PU-H71 inhibition of Hsp90 also inhibits the MAPK signaling pathway, consequently suppressing the transcription of genes associated with cell survival, angiogenesis, proliferation, migration, mitosis, and differentiation. Experiments also revealed that expression levels of Hsp90 remained constant with varying PU-H71 concentrations but Hsp70 levels increased in response to Hsp90 inhibition at concentrations of approximately 0.1–0.25 µM.

### Biodistribution Studies Show Specific Uptake of ^89^Zr-DFO-Trastuzumab in BT-474 Tumors

**Figure 3:**
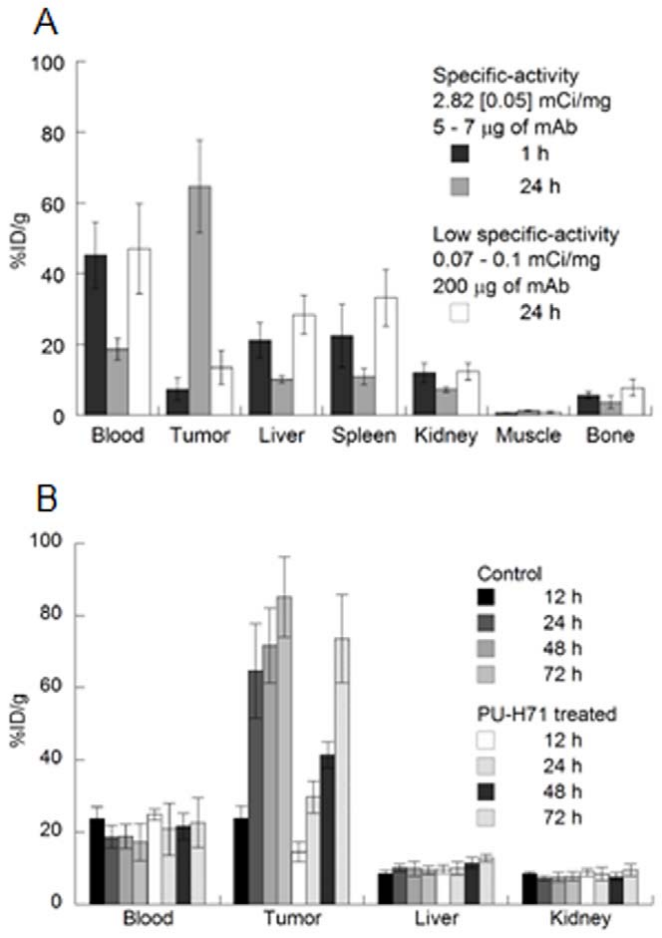
Bar charts showing selected tissue biodistribution data (%ID/g) for (A) uptake of high and low specific-activity formulations of ^89^Zr-DFO-trastuzumab in BT-474 tumor-bearing mice, and (B) ^89^Zr-DFO-trastuzumab uptake in control (vehicle-treated) and PU-H71 treated animals at 12, 24, 48 and 72 h post-i.v. administration of ^89^Zr-DFO-trastuzumab (0.55–0.74 MBq, 5–7 µg of mAb, in 200 µL 0.9% sterile saline).

The ability of ^89^Zr-DFO-trastuzumab to target HER2/*neu* receptors *in vivo* was initially assessed by conducting acute biodistribution studies in BT-474 tumor-bearing mice at 1, 12, 24, 48, 72 and 120 h post-i.v. administration (Tables 1 and S1, and Figure 3). The data reveal that high tumor uptake is observed 24 h post-injection (64.68±13.06%ID/g) with ^89^Zr accumulation in the tumors reaching a peak between 48 and 72 h. Retention of ^89^Zr was observed for over 120 h (75.54±13.47%ID/g) with only a small decrease (9.64%ID/g, [*P* = 0.28]) in the mean uptake compared to that at 72 h.

**Table 1:** Biodistribution data of ^89^Zr-DFO-trastuzumab *versus* time/h, administered by i.v. tail-vein injection to female, athymic *nu*/*nu* mice bearing s.c. BT-474 tumors (90–150 mm^3^).a,b

	^89^Zr-DFO-trastuzumab (5–7 µg mAb)					
Organ	1 h (*n* = 5)	12 h (*n* = 4)	24 h (*n* = 8)	48 h (*n* = 3)	72 h (*n* = 8)	120 h (*n* = 4)
Blood	45.29±9.33	23.70±3.28	18.68±3.12	18.83±3.35	17.23±5.06	11.05±6.98
Tumor	7.35±3.22	23.80±3.37	64.68±13.06	71.71±10.35	85.18±11.10	75.54±13.47
Heart	12.61±3.38	8.22±3.46	5.05±0.90	6.58±1.98	4.75±1.62	3.25±1.35
Lung	18.09±3.48	14.50±2.79	12.36±1.87	13.02±2.72	12.76±3.78	7.11±4.07
Liver	21.18±4.97	8.44±0.98	10.10±1.02	9.96±1.99	9.48±1.13	10.55±2.78
Spleen	22.50±8.89	7.85±1.28	10.92±2.29	6.34±1.69	10.82±3.80	10.85±3.18
Kidney	12.02±2.72	8.43±0.63	7.28±0.66	7.59±1.21	7.75±1.23	6.48±1.67
Muscle	0.68±0.08	1.36±0.38	1.22±0.18	1.12±0.26	1.25±0.40	1.03±0.42
Bone	5.64±0.90	1.93±0.71	3.67±1.71	1.68±0.47	4.79±0.65	6.86±3.02
Tumor/Blood	0.17±0.09	1.06±0.23	3.50±0.78	3.91±1.04	5.18±1.90	5.68±2.13
Tumor/Heart	0.64±0.32	3.52±2.03	12.94±2.75	11.71±4.44	19.59±6.30	20.26±5.81
Tumor/Lung	0.44±0.27	1.80±0.72	5.25±0.90	5.75±1.90	7.09±2.47	9.27±3.62
Tumor/Liver	0.36±0.14	2.86±0.74	6.39±1.11	7.37±1.54	9.44±1.98	8.20±1.54
Tumor/Spleen	0.33±0.09	2.93±0.88	5.99±1.04	11.84±3.25	9.21±2.59	8.06±1.48
Tumor/Kidney	0.66±0.36	2.95±0.62	8.83±1.36	9.65±2.33	10.98±1.59	10.54±2.40
Tumor/Muscle	11.30±5.80	18.34±9.95	54.27±5.07	67.80±4.51	75.81±6.63	67.67±5.94
Tumor/Bone	1.32±0.55	10.01±1.50	9.63±6.73	45.55±16.54	18.41±4.90	14.21±2.79

	Block (200 µg mAb)		PU-H71 treated (5–7 µg mAb)			
Organ	24 h (*n* = 4)		12 h (*n* = 5)	24 h (*n* = 5)	48 h (*n* = 4)	72 h (*n* = 5)
Blood	47.02±12.72		24.89±1.54	20.79±7.20	21.59±3.67	22.63±6.88
Tumor	13.50±4.84		14.55±2.75	29.75±4.43	41.42±3.64	73.64±12.17
Heart	12.58±3.95		8.36±1.67	5.01±0.63	4.48±1.51	5.69±0.45
Lung	25.28±7.34		15.64±1.85	12.84±5.39	13.55±3.82	13.16±3.45
Liver	28.37±5.49		9.74±1.20	10.02±1.80	11.46±1.52	12.89±1.04
Spleen	33.22±8.01		9.94±2.01	11.20±2.52	10.15±2.44	12.66±4.92
Kidney	12.33±2.39		8.89±1.00	8.46±1.81	7.64±1.07	9.55±1.59
Muscle	0.87±0.17		1.18±0.15	1.27±0.23	0.97±0.28	1.55±0.40
Bone	7.79±2.39		2.66±0.99	5.09±2.32	2.23±0.47	5.27±1.48
Tumor/Blood	0.33±0.20		0.55±0.09	1.53±0.51	1.96±0.23	3.36±0.52
Tumor/Heart	1.28±0.89		1.76±0.82	6.22±1.56	8.16±1.09	12.89±1.16
Tumor/Lung	0.67±0.23		0.89±0.06	2.74±1.28	3.55±0.64	5.70±0.61
Tumor/Liver	0.48±0.30		1.43±0.05	3.27±0.77	3.79±0.39	5.78±1.36
Tumor/Spleen	0.50±0.20		1.36±0.02	2.82±0.85	4.68±0.18	6.54±2.81
Tumor/Kidney	1.17±0.56		1.49±0.30	3.54±0.44	5.16±0.13	7.71±0.28
Tumor/Muscle	16.61±7.61		12.54±1.35	24.90±2.74	36.93±1.47	48.47±5.39
Tumor/Bone	1.90±1.03		4.64±0.92	7.86±3.86	21.17±5.14	14.98±5.44

^a^Complete biodistribution data are presented in the supporting information (Table S1).

^b^The data are expressed as the mean %ID/g ± one standard deviation (S.D.).

Competitive inhibition studies using low specific-activity formulations (30–40-fold decrease: 2.60–3.65 MBq/mg [0.07–0.1 mCi/mg]) revealed only 13.50±4.84%ID/g tumor uptake at 24 h post-injection; an approximate 5-fold decrease (*P*<0.00001) *versus* high specific-activity formulations (Figure 3A). These experiments concur with the *in vitro* data and demonstrate the specificity of ^89^Zr-DFO-trastuzumab for the HER2/*neu* antigen *in vivo*.

**Figure 4:**
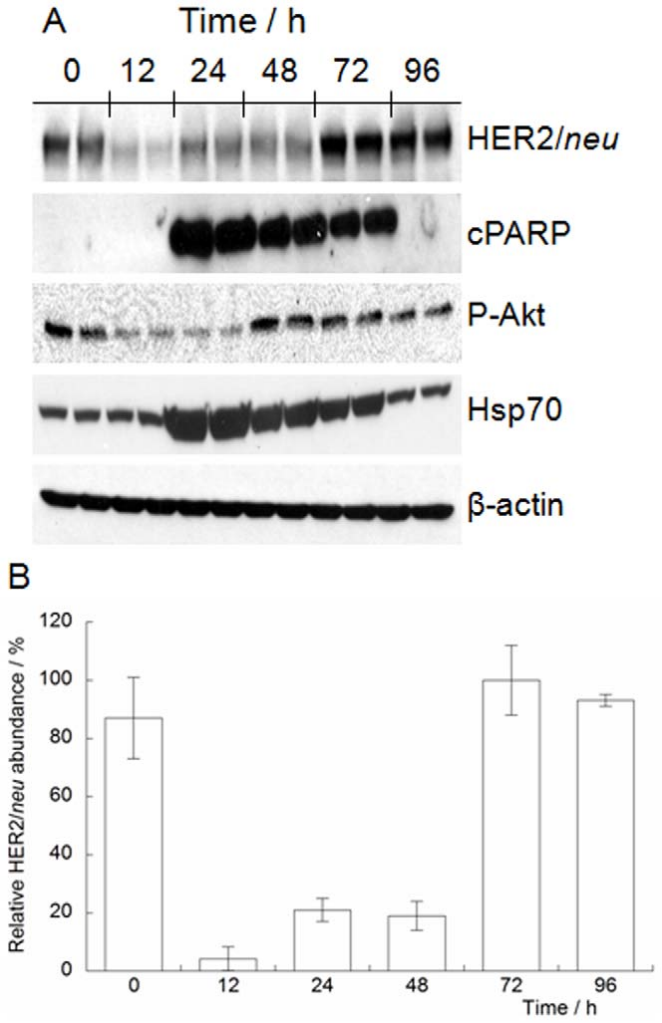
Pharmacodynamic studies on protein expression levels in BT-474 tumor tissue samples obtained at 12, 24, 48, 72 and 96 h after PU-H71 treatment.

The pharmacodynamic effects of administering PU-H71 on ^89^Zr-DFO-trastuzumab uptake in BT-474 tumor-bearing mice were also investigated by using acute biodistribution studies (Table 1 and S1, and Figure 3B) and Western blot analysis of tissue samples (Figure 4). Animals were treated with a single dose of PU-H71 4 h prior to i.v. injection of ^89^Zr-DFO-trastuzumab (*t* = 0 h) and biodistribution studies were conducted at 12, 24, 48 and 72 h. The biodistribution data show that at 24 and 48 h post-injection, tumor uptake in PU-H71-treated mice reached 29.75±4.43%ID/g and 41.42±3.64%ID/g, respectively. Thus, in comparison with control experiments, PU-H71 inhibition of Hsp90 and degradation of HER2/*neu* results in approximately 50% decrease in ^89^Zr-DFO-trastuzumab tumor uptake at 24 and 72 h (Table S2; Control-to-PU-H71-treated %ID/g ratios of 2.17 [*P* = 0.0001] and 1.73 [*P* = 0.026], respectively). By 72 h post-administration, ^89^Zr-DFO-trastuzumab tumor uptake in PU-H71-treated mice (73.64±12.17%ID/g) returned to the same levels as observed in the control experiments (85.18±11.10%ID/g, [*P* = 0.244]). Importantly, ^89^Zr-DFO-trastuzumab uptake in all background tissues remained the same in control and PU-H71 treated animals. This observation demonstrates the specificity of PU-H71 for HER2/*neu* over-expressing BT-474 tumors.

The ^89^Zr-DFO-trastuzumab biodistribution data suggest that a single-dose of PU-H71 induces a sustained effect in terms of HER2/*neu* and other client oncoprotein degradation for at least 48 h post-administration. This observation was confirmed by conducting Western blot analysis for HER2/*neu* expression in homogenized tumor tissue samples obtained at 0, 12, 24, 48, 72 and 96 h post-i.p. administration of PU-H71 in BT-474 tumor-bearing mice (Figures 4A and B). As with the *in vitro* experiments, PU-H71 was found to inhibit Hsp90 as indicated by the specific tumor fingerprints which showed increased Hsp70 expression, degradation of P-Akt and high levels of cPARP between 24 and 72 h. The data confirm that PU-H71 degrades HER2/*neu* levels to ∼20% of initial levels for up to 48 h. HER2/*neu* expression was found to return to baseline levels by 72 h. The recovery of HER2/*neu* expression between 48 and 72 h is consistent with the increase in ^89^Zr-DFO-trastuzumab uptake observed in the biodistribution studies.

### ImmunoPET Studies Demonstrate the Use of ^89^Zr-DFO-Trastuzumab as a Radiotracer

**Figure 5:**
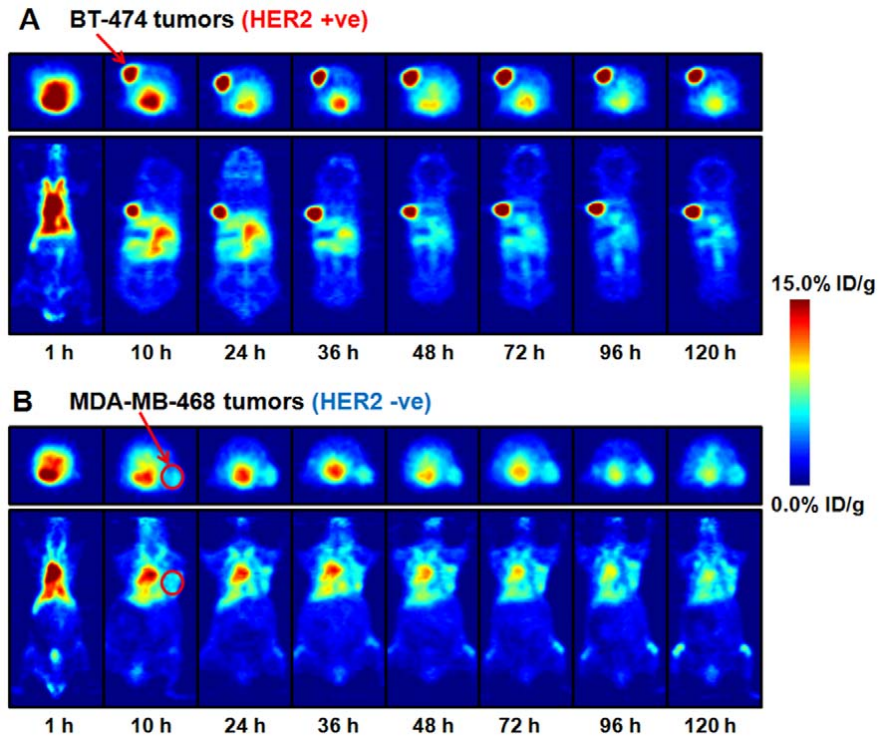
ImmunoPET images of ^89^Zr-DFO-trastuzumab (8.50–9.25 MBq, 80–90 µg of mAb, in 200 µL 0.9% sterile saline) recorded in (A) BT-474 (left shoulder) and (B) MDA-MB-468 (right flank) tumor-bearing mice between 1–120 h post-injection. The transverse (top) and coronal (bottom) planar images intersect the center of the tumors.

**Figure 6:**
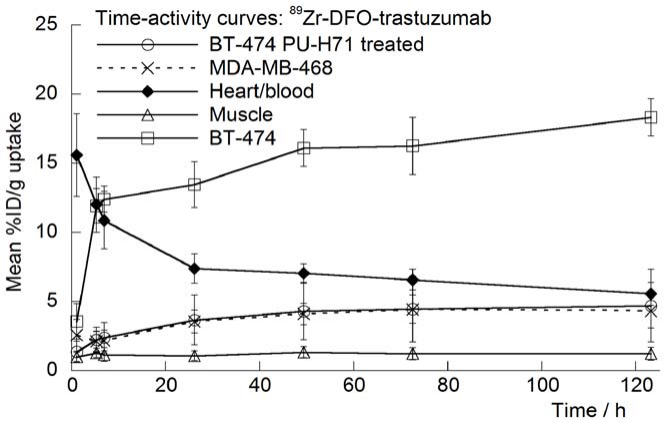
Time-activity curves derived by region-of-interest analysis of the immunoPET images showing the mean %ID/g tissue uptake *versus* time/h, for control and PU-H71-treated mice bearing both BT-474 and MDA-MB-468 tumors.

ImmunoPET images of ^89^Zr-DFO-trastuzumab recorded in BT-474 and MDA-MB-468 tumor-bearing mice between 1 to 120 h are presented in Figures 5A and B, respectively. Radiotracer uptake in BT-474 tumors was observed in <5 h post-injection of ^89^Zr-DFO-trastuzumab and high tumor-to-muscle (T/M) ratios (calculated by using both region-of-interest mean and maximum %ID/g values from the PET images; Figure 6 and Table S3) were observed. At 5 h post-injection mean and maximum T/M ratios for radiotracer uptake in BT-474 tumor-bearing mice were found to be 7.87 and 11.90, respectively. In contrast, low ^89^Zr-DFO-trastuzumab accumulation in HER2/*neu* negative MDA-MB-468 tumors (mean and maximum T/M ratios of 2.26 and 4.15, respectively) was found to occur in accordance with the “enhanced permeation and retention” (EPR) mechanism.[58]

Quantification of the immunoPET images showed that at 48 and 72 h time points, the absolute uptake of ^89^Zr-DFO-trastuzumab was around 16.0%ID/g in BT-474 tumors and 4.5%ID/g in MDA-MB-468 tumors (Figure 6). These data show that ^89^Zr-DFO-trastuzumab immunoPET imaging provides very high tumor-to-background tissue (T/B) ratios. Although the models are not identical which prevents direct comparison, the high contrast observed in the immunoPET images with ^89^Zr-DFO-trastuzumab compare favorably with those obtained using other mAb and mAb-fragments labeled with ^64^Cu, ^68^Ga, ^86^Y, ^99m^Tc, ^111^In or ^124^I. [19]–[30] Although the immunoPET images demonstrated very high contrast for radiotracer uptake in BT-474 tumors, quantitative analysis suggests that ^89^Zr-DFO-trastuzumab is approximately 5-fold lower than was observed in the biodistribution experiments. This discrepancy is explained by considering the difference in the amount of mAb administered between the two experiments. For the immunoPET experiments a total of 80–90 µg of trastuzumab was administered, whereas in the biodistribution assays only 5–7 µg was used. The use of different total doses of trastuzumab is unavoidable given the limitations in sensitivity of small-animal PET detection which requires larger amounts of administered activity than simple biodistribution experiments. In humans undergoing trastuzumab therapy, the mAb is administered at concentrations >5 mg/kg.[59] Based on the human values, a therapeutic dose of trastuzumab in a typical 20–25 g mouse, corresponds to 100–125 µg of mAb. Consequently, the specific-activity of ^89^Zr-DFO-trastuzumab becomes a factor in determining the precise correlation between the uptake values obtained from the biodistribution and quantitative immunoPET images. In other words, at the specific-activity obtained, the amount of mAb administered in the immunoPET experiments causes approximately 80% competitive binding (blocking) of available HER2/*neu* antigens with non-radiolabeled trastuzumab. Importantly, previous studies in our laboratory using the Focus 120 microPET and “non-saturating” PET radiotracers have demonstrated close agreement between imaging and biodistribution-derived activity concentrations.

**Figure 7:**
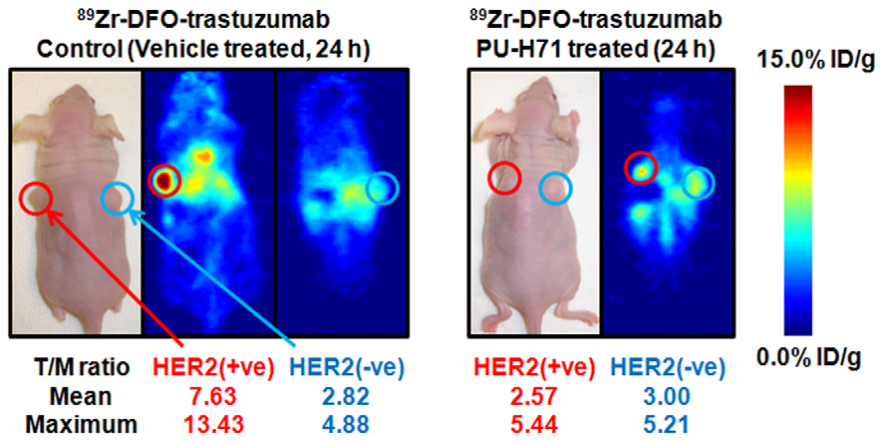
Coronal immunoPET images of control (left) and PU-H71 (right) treated mice bearing s.c. BT-474 and MDA-MB-468 tumors recorded at 24 h post-i.v. administration of ^89^Zr-DFO-trastuzumab. Mean and maximum T/M ratios obtained from VOI analysis are presented (Table S3). For the control animal, the two coronal PET slices lie through the center of the HER2/*neu* positive and negative tumors.

ImmunoPET studies were also used to visualize and quantify the pharmacodynamic effects of PU-H71 treatment in dual tumor-bearing mice. The immunoPET images (Figure 7) and time-activity curves (TACs; Figure 6) show that in PU-H71 treated mice, no difference was observed in either the mean or maximum T/M ratios between BT-474 and MDA-MB-468 tumors. For example, 24 h after administering ^89^Zr-DFO-trastuzumab mean T/M ratios in PU-H71-treated mice were measured as 2.57 and 3.00 in BT-474 and MDA-MB-468 tumors, respectively (Figure 7 and Table S3).

In contrast, large differences were observed in the measured T/M ratios between BT-474 (T/M = 7.63) and MDA-MB-468 (T/M = 2.82) tumors in vehicle-treated mice. At longer time points, mean T/M ratios for BT-474 tumors in vehicle-treated mice remained constant at 48 h (7.85) but decreased slightly by 72 h (6.71) and 120 h (6.77).

The approximate 3-fold decrease in BT-474 T/M ratios between the vehicle-treated and PU-H71 treated mice is consistent with the decrease in HER2/*neu* expression levels observed by *in vivo* Western blots (Figure 4). Furthermore, the slight decrease in tumor uptake between 72 and 120 h is consistent with the 9.6%ID/g decrease in BT-474 tumor uptake observed from the biodistribution studies. This decreased tumor uptake at longer time points is potentially indicative of a slow rate of metabolism and excretion of ^89^Zr^4+^ after internalization of HER2/*neu*-bound ^89^Zr-DFO-trastuzumab (Figures S4 and S5, and Table S4).

At late time points, the biodistribution data for ^89^Zr-DFO-trastuzumab show residual uptake in the bone (4.79±0.65%ID/g and 6.86±3.02%ID/g at 72 and 120 h, respectively). This bone uptake was confirmed by immunoPET, and images of ^89^Zr-DFO-trastuzumab at 120 h displayed low liver uptake but slightly higher activity in the joints of the hind legs. It is plausible that a slow rate of intratumoral metabolism of ^89^Zr-DFO-trastuzumab leads to the transmetalation of ^89^Zr^4+^ ions to a species which is either excreted or sequestered in bone. However, our stability studies and biodistribution data, along with previous reported investigations on other ^89^Zr-DFO-labeled antibodies suggest that metabolism is very limited.[44]–[46] Full metabolism studies are beyond the scope of this work but further investigations on the fate of ^89^Zr-DFO-trastuzumab *in vivo* are underway.

The studies reported here are consistent with previous investigations and confirm that ^89^Zr is a suitable radionuclide for labeling full, intact antibodies (∼150 kDa).[30] The favorable chemistry and image characteristics mean that ^89^Zr has the potential to solve problems associated with, for example, non-optimal half-life (^64^Cu, ^86^Y), high uptake in background tissue (^64^Cu, ^68^Ga, ^124^I), low *in vivo* stability (^124^I; particularly with internalizing antibody-antigen constructs) and poor dosimetry (^86^Y). Furthermore, these studies demonstrate that immunoPET with ^89^Zr-labeled mAbs can be used for both localizing tumors and measuring the long-term effects (>5 days) of drug treatment from a single radiotracer injection. Such measurements cannot be achieved by using previous ^64^Cu-, ^86^Y-, ^99m^Tc-, and ^124^I-radiolabeled trastuzumab constructs. Along with ^18^F-labeled trastuzumab affibodies[29], ^89^Zr-DFO-trastuzumab represents one of the most promising radiotracers for non-invasive immunoPET measurements of HER2/*neu* expression *in vivo*. These studies also showed that, unlike most tyrosine-kinase inhibitors, PU-H71 displays an extended pharmacodynamic profile for Hsp90 inhibition at time points >24 h, and this window of activity is ideal for immunoPET imaging with radioimmunoconjugates such as ^89^Zr-DFO-trastuzumab. As the pharmacodynamic change in HER2/*neu* expression levels is indicative of the potential anti-tumor effects of PU-H71, ^89^Zr-DFO-trastuzumab may also be used to further the clinical development of this, and related Hsp90 inhibitors, by evaluating the dose delivered to the tumor and quantifying the magnitude of the therapeutic response. These immunoPET studies demonstrate that ^89^Zr-DFO-trastuzumab has the potential to be used in the clinic as a specific radiotracer for measuring the long-term effects of treatment with Hsp90 inhibitors.

In conclusion, ^89^Zr-DFO-trastuzumab has been prepared with high radiochemical purity and specific-activity using methods adapted from the literature. The immunoconjugate was found to be stable with respect to loss of the radiolabel *in vitro* and immunoreactivity assays demonstrated that conjugation, radiolabeling and purification chemistries do not compromise the binding affinity or specificity for the HER2/*neu* antigen. Biodistribution and immunoPET experiments indicated that ^89^Zr-DFO-trastuzumab shows excellent potential as a radiotracer for specific non-invasive delineation of HER2/*neu* positive breast tumors *in vivo*. Furthermore, unlike other trastuzumab constructs radiolabeled with shorter-lived nuclides, ^89^Zr-DFO-trastuzumab may be used to measure the long-term pharmacodynamic effects and potentially patient response to treatment using Hsp90 inhibitors including PU-H71. Work towards the clinical translation of ^89^Zr-DFO-trastuzumab and other ^89^Zr-labeled mAbs is underway.

## Supporting Information

Text S1 Supplementary Materials and Methods.
(0.07 MB DOC)

Figure S1 Typical elution profiles observed by using PD-10 size-exclusion chromatography for the purification of ^89^Zr-DFO-trastuzumab from small molecule (<30 kDa) ^89^Zr-radiolabeled impurities and unreacted [^89^Zr]Zr-oxalate (complexed as [^89^Zr]Zr-DTPA). Species with molecular weights >30 kDa elute in the first <2.0 mL of solvent. The two peaks for crude and purified ^89^Zr-DFO-trastuzumab have the same retention time within the full-width half-maximum (FWHM).
(0.14 MB TIF)

Figure S2 (A) Competitive inhibition (blocking) studies. (B) Cellular association with MDA-MB-468 (HER2/neu -ve) cells.
(0.25 MB TIF)

Figure S3 Chemical structure of PU-H71
(0.05 MB TIF)

Figure S4 Plot of the total normalized, average number of coincident counts recorded via immunoPET imaging of BT-474 tumor-bearing mice (n = 4) versus time/h. Exponential decay regression analysis has been used to calculate the effective lifetime (τ_eff_/h) and decay constant (λ_eff_/h^−1^) from which the estimated observed and biological half-lives (t_1/2.eff_ and t_1/2.biol_) have been calculated.
(0.13 MB TIF)

Figure S5 Plot of the total normalized, average number of coincident counts recorded via immunoPET imaging of MDA-MB-468 tumor-bearing mice (n = 4) versus time/h. Exponential decay regression analysis has been used to calculate the effective lifetime (τ_eff_/h) and decay constant (λ_eff_/h^−1^) from which the estimated observed and biological half-lives (t_1/2.eff_ and t_1/2.biol_) have be calculated.
(0.13 MB TIF)

Table S1 Biodistribution data of ^89^Zr-DFO-trastuzumab versus time/h, administered by i.v. tail-vein injection to female, athymic nu/nu mice bearing s.c. BT-474 tumors (90–150 mm^3^).^a^
(0.11 MB DOC)

Table S2 A comparison of the difference between mean BT-474 tumor uptake values observed in the biodistribution studies in control (vehicle-treated) and PU-H71 treated mice.
(0.04 MB DOC)

Table S3 Tumor-to-muscle (T/M) ratios have been calculated from volume-of-interest (VOI) analysis of immunoPET images recorded in dual tumor-bearing (BT-474 and MDA-MB-468) female, athymic nu/nu mice between 1–120 h post-i.v. administration of ^89^Zr-DFO-trastuzumab. The data presented are ratios of mean and maximum (%ID/g) values. Errors associated with VOI activity measurements are large and are strongly dependent on the number and definition of each ROI used in the determination of the VOI. As a consequence of the large errors associated with VOI analysis, and the further exaggeration that ensues from the calculation of ratios, errors associated with the calculated ratios are large, difficult to define and have been omitted to avoid misrepresentation of the data.
(0.05 MB DOC)

Table S4 Summary of the calculated effective and biological half-lives
(0.04 MB DOC)
