# Supplementary material for: Measuring the Pharmacodynamic Effects of a Novel Hsp90 Inhibitor on HER2/neu Expression in Mice Using 89Zr-DFO-Trastuzumab
Source: PLoS One. 2010 Jan 25;5(1):e8859. doi: 10.1371/journal.pone.0008859 (PMC2810330; doi:10.1371/journal.pone.0008859)
Supplement: Text S1 — Supplementary Materials and Methods. (0.07 MB DOC) [file pone.0008859.s001.doc]

## Trastuzumab conjugation

*Synthesis of N-succinyldesferrioxamine B (N-succDFO).* DFO mesylate (0.508 g, 0.77 mmol, Calbiochem, Spring Valley, CA) was dissolved in pyridine (7.5 mL) and reacted with excess succinic anhydride (1.704 g, 0.017 mol) at room temperature for 24 h. The white suspension was then poured into NaOH(aq.) (120 mL, 0.015 mol dm^-3^) and stirred at room temperature for 16 h. The colorless solution was adjusting to pH2 by the addition of 12 mol dm^-3^ HCl and cooled with stirring at 4 ºC for 2 h. The white precipitate was collected by filtration, washed with copious amounts of HCl (0.01 mol dm^-3^) then water and dried *in vacuo* to give the *N*-succinyldesferrioxamine B (*N*-succDFO) as a white microcrystalline solid (0.306 g, 4.75 × 10^-4^ mol, 62%). HRMS-ES^+^: Calc. for [C_29_H_52_N_6_O_11_ + H^+^] = 661.3772; found 661.3760 ([M + H^+^] = 100%).

*Preparation of [Fe(N-succDFO-TFP)] activated ester.* *N*-succDFO (9.0 mg, 14 µmol) was suspended in 3.0 mL 0.9% sterile saline and the pH adjusted to 6.5 with 0.1 M Na_2_CO_3_(aq.) (50 – 75 µL in chelex purified water). Then a solution of FeCl_3_•6H_2_O [4.0 mg, 15 µmol, 300 µL of 0.1 M HCl(aq.)] was added. Upon addition of the FeCl_3_(aq.) the reaction mixture changes from colorless to deep orange due to the intense electronic absorption band of [Fe(DFO)] with a peak at 430 nm (ε_430_ = 2216 ± 49 mol^-1^ dm^3^ cm^-1^). After stirring the reaction at room temperature for 1 h, a solution of 2,3,5,6-tetrafluorophenol (TFP, 300 µL, 36 µmol, 1.2 mol dm^-3^ in chelex purified MeCN; SigmaAldrich, St. Louis, MO) was added to the reaction followed by addition of solid *N*-(3-dimethylaminopropyl)-*N’*-ethylcarbodiimide hydrochloride (EDAC, 120 mg, 0.63 mmol, SigmaAldrich) The reaction mixture (pH6.5) was then stirred at room temperature for 1 h before purifying the [Fe(*N*-succDFO-TFP)] product by use of a C-18 Light Sep-pak cartridge (Waters, Milford, MA). The reaction mixture was loaded onto a pre-activated (6 mL MeCN, 10 mL H_2_O) C-18 cartridge, washed with copious amounts of water (>40 mL), and eluted with 1.5 mL MeCN. The final [Fe(*N*-succDFO-TFP)] solution had a concentration approximately 9.3 mM. The [Fe(*N*-succDFO-TFP)] solution can be stored for 24 h at 4 ºC but the most efficient conjugation reactions were achieved by using fresh preparations.

*Preparation of DFO-trastuzumab.* Trastuzumab (20 mg, 0.137 µmol, MW 145591.5 g mol^-1^, Herceptin^TM^, Genentech, South San Francisco, CA) was dissolved in 1.0 mL of chelex purified water) and the pH adjusted to 9.5 – 10.0 by using aliquots of 0.1 M Na_2_CO_3_(aq.) (approx. 50 µL total). A 10-fold excess of [Fe(*N*-succDFO-TFP)] (1.37 µmol, 147 µL) was added with gentle mixing using an automated pipette, and the reaction was allowed to react at room temperature without agitation for 1 h. Then 2,5-dihydroxybenzoic acid [gentisic acid, 50 µL, 0.65 mol dm^-3^ in 0.32 M Na_2_CO_3_(aq.)] was added to the reaction and the pH was adjusted to 3.9 – 4.2 by the addition of 5 to 10 µL aliquots of 0.25 M H_2_SO_4_(aq.). Then a 10-fold excess of ethylenediaminetetraacetic acid disodium salt with [EDTA^2-^.2Na^+^(aq.), 0.0674 mol dm^-3^, 13.7 µmol, 200 µL] with respect to [Fe(*N*-succDFO-TFP)] was added. The reaction was incubated in a water bath at 38 ºC for 1 h during which time the solution changed from clear yellow to colorless. NB: in various preparations of the DFO functionalized trastuzumab, a slightly cloudy appearance of the solution was observed. This is likely due to precipitation of the mAb at low pH. Subsequent purification led to colorless solutions which were found to have immunoreactive fractions >0.8. The DFO-trastuzumab was purified by either size-exclusion chromatography (Sephadex G-25 M, PD-10, >30 kDa, GE Healthcare; dead-volume = 2.5 mL, eluted with 200 µL fractions of 0.9% sterile saline) or spin-column centrifugation (4 mL volume, >30 kDa, Amicon Ultra-4, Millipore, Billerica, MA; washed with 4 × 3 mL, 0.9% sterile saline) in accordance with the manufacturers’ instructions. Spin-column centrifugation was found to be more efficient with reduced protein losses and higher concentrations of the final DFO-trastuzumab product.

^89^Zr-DFO-trastuzumab was prepared in accordance with the procedures described in the main text. Figure S1 shows a typical elution profile for the purification of ^89^Zr-DFO-trastuzumab by using PD-10 size-exclusion chromatography.

## Cell Lines

Human breast cancer cell lines BT-474 and MDA-MB-468 were obtained from the American Type Culture Collection (ATCC, Manassas, VA). Cells were grown by weekly serial passage in a 1:1 mixture of Dulbecco’s Modified Eagle medium/F-12 medium, supplemented with 10% heat inactivated fetal calf serum (FCS, Omega Scientific, Tarzana, CA), 2.0 mM glutamine, non-essential amino acids, and 100 units/mL of both penicillin and streptomycin in a 5% CO_2_(g) atmosphere at 37 ºC. Cells were harvested by using a formulation of 0.25% Trypsin and 0.53 mM EDTA in Hank’s Buffered Salt Solution (HBSS) without calcium or magnesium.

## Chelate number

The number of accessible DFO chelates conjugated to trastuzumab was measured radiometric by isotopic dilution assays following a method similar to that described by Anderson *et al*.[1,2] Aliquots of [^89^Zr]Zr-oxalate (10 µL, <5 kBq [0.1 µCi]) were added to 12 solutions containing 1:2 serial dilutions of non-radioactive ZrCl_4_(aq.) (100 µL fractions; 100 – 0.05 nmol, pH7.5 – 8.5). The mixture was vortexed for 30 s before adding aliquots of DFO-trastuzumab (20 µL, 5.0 mg/mL, [100 µg of mAb, 0.7 nmol], in 0.9% sterile saline). The reactions were incubated at room temperature for 1 h before quenching with DTPA (20 µL, 50 mM pH7). The extent of complexation was assessed developing ITLC strips (DTPA, 50 mM) and counting the activity at the baseline and solvent front. The fraction of ^89^Zr-radiolabeled mAb (*A_b_*) was plotted *versus* the inverse of the number of nano-moles of Zr and the number of chelates was calculated from the gradient by Equation S1, where *c* is the number of accessible chelates, *n*(mAb) is the amount of mAb (nmol), and *n*(Zr) is the total number of Zr added (nmol).[3]

| $c=A_{b}\cdot\frac{n\left(\text{Zr}\right)}{n\left(\text{mAb}\right)}$ | (S1) |
| --- | --- |

## Small-animal immunoPET imaging

PET imaging experiments were conducted on a microPET Focus 120 scanner (Concorde Microsystems).[4] Mice were administered ^89^Zr-DFO-trastumab formulations (8.50 – 9.25 MBq, [230 – 250 µCi], 80 – 90 µg of mAb, in 200 µL 0.9% sterile saline for injection) *via* i.v. tail-vein injection. Approximately 5 minutes prior to recording PET images, mice were anesthetized by inhalation of 1% isoflurane (Baxter Healthcare, Deerfield, IL)/oxygen gas mixture and placed on the scanner bed. PET images were recorded at various time-points between 1 – 120 h. List-mode data were acquired for between 10 and 30 min. using a γ-ray energy window of 350 – 750 keV, and a coincidence timing window of 6 ns. For all static images, a minimum of 20 million coincident events were recorded. Data were sorted into 2-dimensional histograms by Fourier re-binning, and transverse images were reconstructed by filtered back-projection (FBP) into a 128 × 128 × 63 (0.72 × 0.72 × 1.3 mm) matrix. The reconstructed spatial resolution for ^89^Zr was 1.9 mm full-width half maximum (FWHM) at the center of the field-of-view (FOV). The image data were normalized to correct for non-uniformity of response of the PET, dead-time count losses, positron branching ratio, and physical decay to the time of injection but no attenuation, scatter, or partial-volume averaging correction was applied. An empirically determined system calibration factor (in units of [mCi/mL]/[cps/voxel]) for mice was used to convert voxel count rates to activity concentrations. The resulting image data were then normalized to the administered activity to parameterize images in terms of %ID/g. Manually drawn 2-dimensional regions-of-interest (ROIs) or 3-dimensional volumes-of-interest (VOIs) were used to determined the maximum and mean %ID/g (decay corrected to the time of injection) in various tissues.[5] Images were analyzed by using ASIPro VM^TM^ software (Concorde Microsystems).

## Immunoreactivity assays

The immunoreactive fraction of ^89^Zr-DFO-trastuzumab was assessed by using specific, infinite-antigen cellular binding assays similar to the methods described by Lindmo *et al*.[6,7] Figures S2A and S2B demonstrate that cellular binding of ^89^Zr-DFO-trastuzumab is specific for expression of the HER2/*neu* antigen and that cellular binding to the MDA-MB-468 (HER2/*neu* negative) control cell line is very low. No weighting was applied to the data points. Data points are the mean of triplicate samples.

## Xenograft models

All animal experiments were conducted in compliance with Institutional Animal Care and Use Committee (IACUC) guidelines. Female athymic *nu*/*nu* mice (NCRNU-M, 20 – 25 g, 6 – 8 weeks old) were obtained from Taconic Farms Inc. (Hudson, NY), and were allowed to acclimatize at the MSKCC vivarium for 1 week prior to implanting tumors. Mice were provided with food and water *ad libitum*. Tumors were induced on a shoulder by sub-cutaneous (s.c.) injection of 5.0 – 10.0 (× 10^6^) cells in a 200 μL cell suspension of a 1:1 v/v mixture of media with reconstituted basement membrane (BD Matrigel^TM^, Collaborative Biomedical Products Inc., Bedford, MA).[8] For animals bearing two tumors used in the immunoPET studies, BT-474 and MDA-MB-468 cells were implanted s.c. on the left and right shoulder/flank, respectively. Tumors developed after a period of 14 – 18 days and the tumor volume (*V* / mm^3^) was estimated by external vernier caliper measurements of the longest axis, *a* / mm, and the axis perpendicular to the longest axis, *b* / mm. The tumors were assumed to be spheroidal and the volume was calculated in accordance with Equation S2.[9]

## ^89^Zr-DFO-trastuzumab: Estimated biological and effective half-lives

These data are presented to provide an estimate of the effective (observed) and biological half-life of ^89^Zr-DFO-trastuzumab in either BT-474 or MDA-MB-468 tumor-bearing mice. We acknowledge that large errors are likely (particularly in the calculation of the biological half-life, t_1/2.biol_). Nevertheless, these data facilitate the generation of hypotheses related to the natural excretion of ^89^Zr-DFO-trastuzumab from circulation and active metabolism/excretion of ^89^Zr-DFO-trastuzumab in HER2/*neu* positive BT-474 tumors.

The data presented in (Figures S4 and S5, and Table S4) support the conclusion that slow metabolic excretion of ^89^Zr-radioactivity occurs in BT-474 tumor-bearing mice. The calculated estimate of *t*_1/2.eff_ in BT-474 mice was 50.5 ± 6.4 h whereas in MDA-MB-468 mice a higher value of 60.1 ± 8.4 h was observed. The corresponding values of *t*_1/2.biol_ were estimated to be 150.5 ± 49.5 h, and 336.2 ± 184.5 h. We note that the values reported are only crude estimates but nevertheless provide valuable insight into the potential fate of ^89^Zr-DFO-trastuzumab in HER2/*neu* positive tumors.

## References

1. Anderson CJ, Connett JM, Schwarz SW, Rocque PA, Guo LW, et al. (1992) Copper-64-labeled antibodies for PET imaging. J Nucl Med 33: 1685-1690.

2. Anderson CJ, Schwarz SW, Connett JM, Cutler PD, Guo LW, et al. (1995) Preparation, biodistribution and dosimetry of copper-64-labeled anti-colorectal carcinoma monoclonal antibody fragments 1A3-F(ab')_2_. J Nucl Med 36: 850-858.

3. Smith-Jones PM, Fridrich R, Kaden TA, Novak-Hofer I, Siebold K, et al. (1991) Antibody labeling with copper-67 using the bifunctional macrocycle 4-[(1,4,8,11-tetraazacyclotetradec-1-yl)methyl]benzoic acid. Bioconjugate Chem 2: 415-421.

4. Kim JS, Lee JS, Im KC, Kim SJ, Kim S-Y, et al. (2007) Performance measurement of the microPET Focus 120 scanner. J Nucl Med 48: 1527-1535.

5. Tseng J-C, Zanzonico PB, Levin B, Finn R, Larson SM, et al. (2006) Tumor-specific in vivo transfection with HSV-1 thymidine kinase gene using a sindbis viral vector as a basis for prodrug ganciclovir activation and PET. J Nucl Med 47: 1136-1143.

6. Lindmo T, Boven E, Cuttitta F, Fedorko J, Bunn PA, Jr. (1984) Determination of the immunoreactive fraction of radiolabeled monoclonal antibodies by linear extrapolation to binding at infinite antigen excess. J Immunol Methods 72: 77-89.

7. Lindmo T, Bunn PA, Jr. (1986) Determination of the true immunoreactive fraction of monoclonal antibodies after radiolabeling. Methods Enzymol 121: 678-691.

8. Chandarlapaty S, Sawai A, Ye Q, Scott A, Silinski M, et al. (2008) SNX2112, a Synthetic Heat Shock Protein 90 Inhibitor, Has Potent Antitumor Activity against HER Kinase-Dependent Cancers. Clin Cancer Res 14: 240-248.

9. Smith-Jones PM, Solit D, Afroze F, Rosen N, Larson SM (2006) Early tumor response to Hsp90 therapy using HER2 PET: Comparison with ^18^F-FDG PET. J Nucl Med 47: 793-796.
