# Supplementary figures and images for: Measuring the Pharmacodynamic Effects of a Novel Hsp90 Inhibitor on HER2/neu Expression in Mice Using 89Zr-DFO-Trastuzumab
Source: PLoS One. 2010 Jan 25;5(1):e8859. doi: 10.1371/journal.pone.0008859 (PMC2810330; doi:10.1371/journal.pone.0008859)

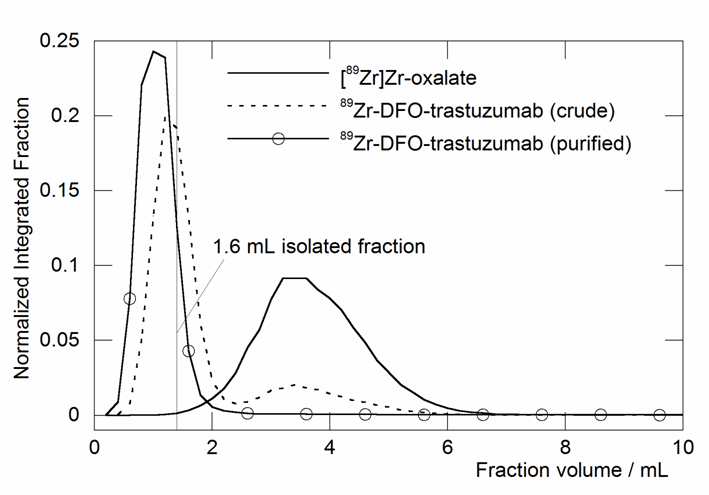

Supplement: Figure S1 — Typical elution profiles observed by using PD-10 size-exclusion chromatography for the purification of 89Zr-DFO-trastuzumab from small molecule (<30 kDa) 89Zr-radiolabeled impurities and unreacted [89Zr]Zr-oxalate (complexed as [89Zr]Zr-DTPA). Species with molecular weights >30 kDa elute in the first <2.0 mL of solvent. The two peaks for crude and purified 89Zr-DFO-trastuzumab have the same retention time within the full-width half-maximum (FWHM). (0.14 MB TIF) [file pone.0008859.s002.tif]

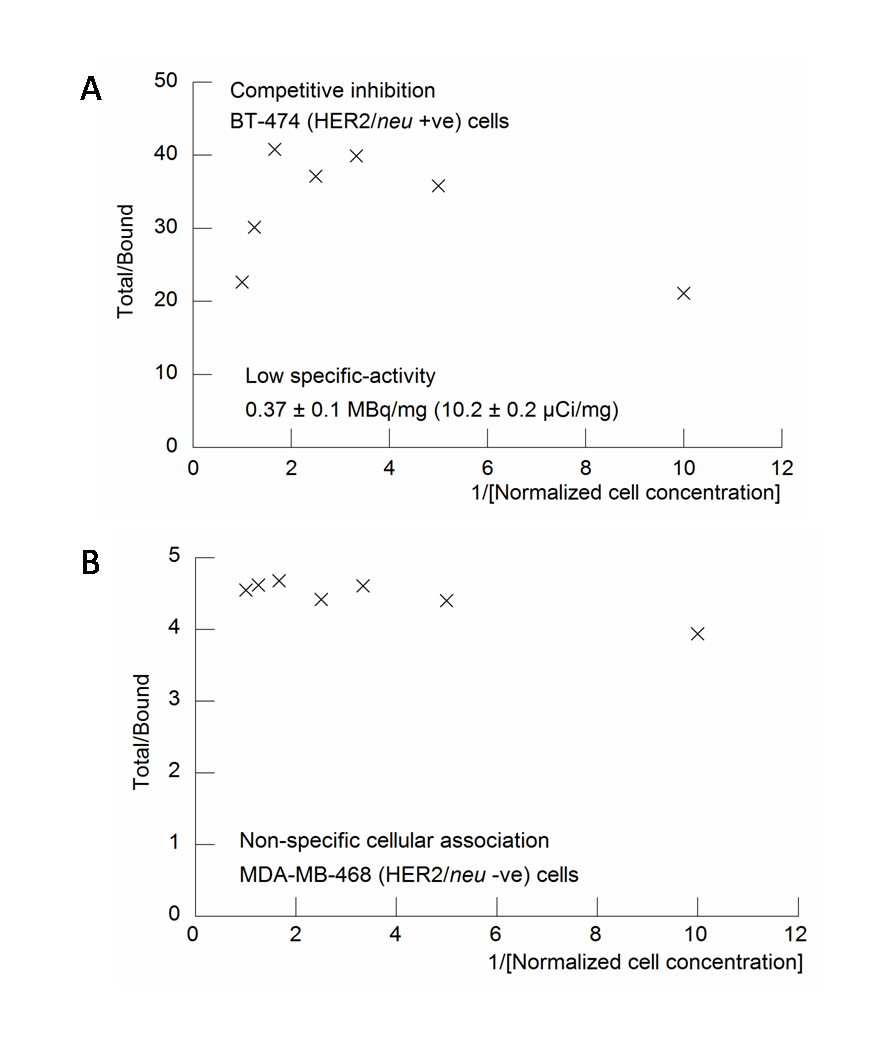

Supplement: Figure S2 — (A) Competitive inhibition (blocking) studies. (B) Cellular association with MDA-MB-468 (HER2/neu -ve) cells. (0.25 MB TIF) [file pone.0008859.s003.tif]

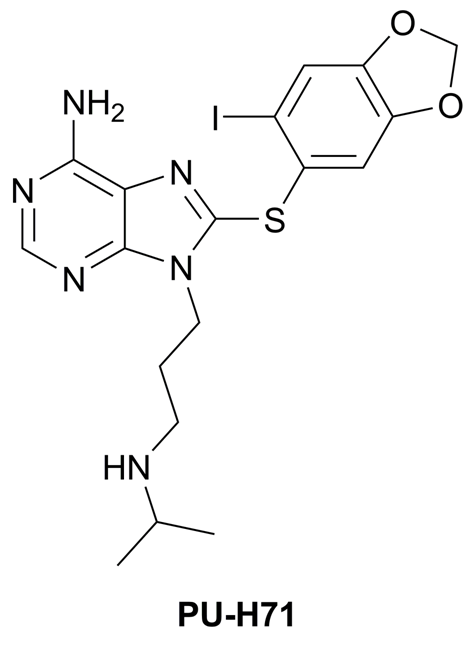

Supplement: Figure S3 — Chemical structure of PU-H71 (0.05 MB TIF) [file pone.0008859.s004.tif]

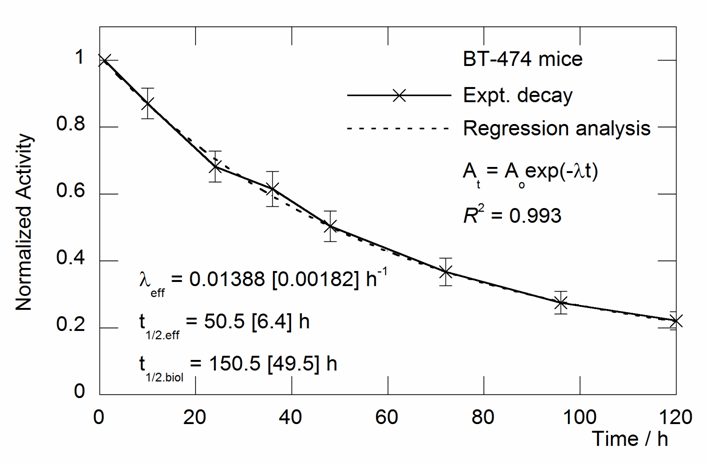

Supplement: Figure S4 — Plot of the total normalized, average number of coincident counts recorded via immunoPET imaging of BT-474 tumor-bearing mice (n = 4) versus time/h. Exponential decay regression analysis has been used to calculate the effective lifetime (τeff/h) and decay constant (λeff/h−1) from which the estimated observed and biological half-lives (t1/2.eff and t1/2.biol) have been calculated. (0.13 MB TIF) [file pone.0008859.s005.tif]

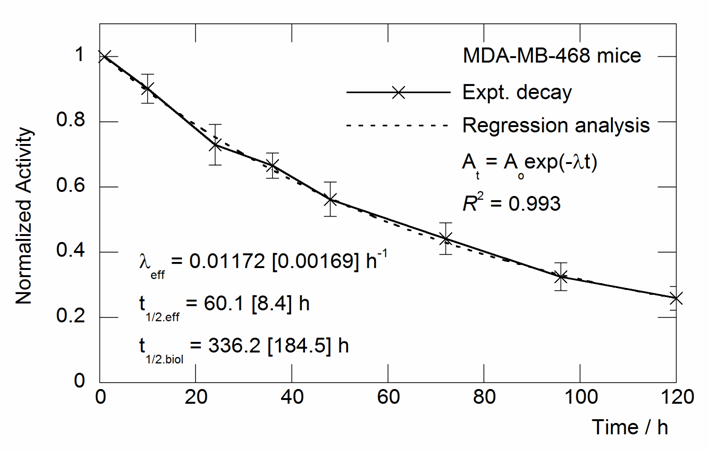

Supplement: Figure S5 — Plot of the total normalized, average number of coincident counts recorded via immunoPET imaging of MDA-MB-468 tumor-bearing mice (n = 4) versus time/h. Exponential decay regression analysis has been used to calculate the effective lifetime (τeff/h) and decay constant (λeff/h−1) from which the estimated observed and biological half-lives (t1/2.eff and t1/2.biol) have be calculated. (0.13 MB TIF) [file pone.0008859.s006.tif]
