# Supplementary material for: Measuring the Pharmacodynamic Effects of a Novel Hsp90 Inhibitor on HER2/neu Expression in Mice Using 89Zr-DFO-Trastuzumab
Source: PLoS One. 2010 Jan 25;5(1):e8859. doi: 10.1371/journal.pone.0008859 (PMC2810330; doi:10.1371/journal.pone.0008859)
Supplement: Table S1 — Biodistribution data of 89Zr-DFO-trastuzumab versus time/h, administered by i.v. tail-vein injection to female, athymic nu/nu mice bearing s.c. BT-474 tumors (90–150 mm3).a (0.11 MB DOC) [file pone.0008859.s007.doc]

|  | **^89^Zr-DFO-trastuzumab** | | | | | |
| --- | --- | --- | --- | --- | --- | --- |
| **Organ** | 1 h (*n* = 5) | 12 h (*n* = 4) | 24 h (*n* = 8) | 48 h (*n* = 3) | 72 h (*n* = 8) | 120 h (*n* = 4) |
| Blood | 45.29 ± 9.33 | 23.70 ± 3.28 | 18.68 ± 3.12 | 18.83 ± 3.35 | 17.23 ± 5.06 | 11.05 ± 6.98 |
| Tumor | 7.35 ± 3.22 | 23.80 ± 3.37 | 64.68 ± 13.06 | 71.71 ± 10.35 | 85.18 ± 11.10 | 75.54 ± 13.47 |
| Heart | 12.61 ± 3.38 | 8.22 ± 3.46 | 5.05 ± 0.90 | 6.58 ± 1.98 | 4.75 ± 1.62 | 3.25 ± 1.35 |
| Lung | 18.09 ± 3.48 | 14.50 ± 2.79 | 12.36 ± 1.87 | 13.02 ± 2.72 | 12.76 ± 3.78 | 7.11 ± 4.07 |
| Liver | 21.18 ± 4.97 | 8.44 ± 0.98 | 10.10 ± 1.02 | 9.96 ± 1.99 | 9.48 ± 1.13 | 10.55 ± 2.78 |
| Spleen | 22.50 ± 8.89 | 7.85 ± 1.28 | 10.92 ± 2.29 | 6.34 ± 1.69 | 10.82 ± 3.80 | 10.85 ± 3.18 |
| Stomach | 2.70 ± 0.96 | 1.10 ± 0.33 | 1.60 ± 0.24 | 1.19 ± 0.74 | 1.40 ± 0.38 | 1.28 ± 0.18 |
| Large Intestine | 0.87 ± 0.39 | 1.23 ± 0.28 | 1.48 ± 0.30 | 1.33 ± 0.55 | 1.27 ± 0.29 | 1.30 ± 0.46 |
| Small Intestine | 5.01 ± 1.18 | 2.59 ± 0.29 | 3.54 ± 1.21 | 2.02 ± 0.44 | 3.40 ± 1.28 | 3.32 ± 1.39 |
| Kidney | 12.02 ± 2.72 | 8.43 ± 0.63 | 7.28 ± 0.66 | 7.59 ± 1.21 | 7.75 ± 1.23 | 6.48 ± 1.67 |
| Muscle | 0.68 ± 0.08 | 1.36 ± 0.38 | 1.22 ± 0.18 | 1.12 ± 0.26 | 1.25 ± 0.40 | 1.03 ± 0.42 |
| Bone | 5.64 ± 0.90 | 1.93 ± 0.71 | 3.67 ± 1.71 | 1.68 ± 0.47 | 4.79 ± 0.65 | 6.86 ± 3.02 |
|  |  |  |  |  |  |  |
| Tumor/Blood | 0.17 ± 0.09 | 1.06 ± 0.23 | 3.50 ± 0.78 | 3.91 ± 1.04 | 5.18 ± 1.90 | 5.68 ± 2.13 |
| Tumor/Heart | 0.64 ± 0.32 | 3.52 ± 2.03 | 12.94 ± 2.75 | 11.71 ± 4.44 | 19.59 ± 6.30 | 20.26 ± 5.81 |
| Tumor/Lung | 0.44 ± 0.27 | 1.80 ± 0.72 | 5.25 ± 0.90 | 5.75 ± 1.90 | 7.09 ± 2.47 | 9.27 ± 3.62 |
| Tumor/Liver | 0.36 ± 0.14 | 2.86 ± 0.74 | 6.39 ± 1.11 | 7.37 ± 1.54 | 9.44 ± 1.98 | 8.20 ± 1.54 |
| Tumor/Spleen | 0.33 ± 0.09 | 2.93 ± 0.88 | 5.99 ± 1.04 | 11.84 ± 3.25 | 9.21 ± 2.59 | 8.06 ± 1.48 |
| Tumor/Kidney | 0.66 ± 0.36 | 2.95 ± 0.62 | 8.83 ± 1.36 | 9.65 ± 2.33 | 10.98 ± 1.59 | 10.54 ± 2.40 |
| Tumor/Muscle | 11.30 ± 5.80 | 18.34 ± 9.95 | 54.27 ± 5.07 | 67.80 ± 4.51 | 75.81 ± 6.63 | 67.67 ± 5.94 |
| Tumor/Bone | 1.32 ± 0.55 | 10.01 ± 1.50 | 9.63 ± 6.73 | 45.55 ± 16.54 | 18.41 ± 4.90 | 14.21 ± 2.79 |

*^a^* The data are expressed as the mean %ID/g ± one standard deviation (S.D.)

**Table S1 continued**

|  | **Block** |  | **PU-H71 treated** | | | |
| --- | --- | --- | --- | --- | --- | --- |
| **Organ** | 24 h (*n* = 4) |  | 12 h (*n* = 5) | 24 h (*n* = 5) | 48 h (*n* = 4) | 72 h (*n* = 5) |
| Blood | 47.02 ± 12.72 |  | 24.89 ± 1.54 | 20.79 ± 7.20 | 21.59 ± 3.67 | 22.63 ± 6.88 |
| Tumor | 13.50 ± 4.84 |  | 14.55 ± 2.75 | 29.75 ± 4.43 | 41.42 ± 3.64 | 73.64 ± 12.17 |
| Heart | 12.58 ± 3.95 |  | 8.36 ± 1.67 | 5.01 ± 0.63 | 4.48 ± 1.51 | 5.69 ± 0.45 |
| Lung | 25.28 ± 7.34 |  | 15.64 ± 1.85 | 12.84 ± 5.39 | 13.55 ± 3.82 | 13.16 ± 3.45 |
| Liver | 28.37 ± 5.49 |  | 9.74 ± 1.20 | 10.02 ± 1.80 | 11.46 ± 1.52 | 12.89 ± 1.04 |
| Stomach | 3.27 ± 1.68 |  | 0.99 ± 0.06 | 1.69 ± 0.35 | 1.34 ± 0.14 | 2.04 ± 0.39 |
| Large Intestine | 1.19 ± 0.41 |  | 1.07 ± 0.21 | 1.67 ± 0.29 | 1.39 ± 0.32 | 1.84 ± 0.36 |
| Small Intestine | 7.47 ± 2.54 |  | 2.80 ± 0.24 | 4.42 ± 1.35 | 2.68 ± 0.32 | 3.61 ± 1.30 |
| Spleen | 33.22 ± 8.01 |  | 9.94 ± 2.01 | 11.20 ± 2.52 | 10.15 ± 2.44 | 12.66 ± 4.92 |
| Kidney | 12.33 ± 2.39 |  | 8.89 ± 1.00 | 8.46 ± 1.81 | 7.64 ± 1.07 | 9.55 ± 1.59 |
| Muscle | 0.87 ± 0.17 |  | 1.18 ± 0.15 | 1.27 ± 0.23 | 0.97 ± 0.28 | 1.55 ± 0.40 |
| Bone | 7.79 ± 2.39 |  | 2.66 ± 0.99 | 5.09 ± 2.32 | 2.23 ± 0.47 | 5.27 ± 1.48 |
|  |  |  |  |  |  |  |
| Tumor/Blood | 0.33 ± 0.20 |  | 0.55 ± 0.09 | 1.53 ± 0.51 | 1.96 ± 0.23 | 3.36 ± 0.52 |
| Tumor/Heart | 1.28 ± 0.89 |  | 1.76 ± 0.82 | 6.22 ± 1.56 | 8.16 ± 1.09 | 12.89 ± 1.16 |
| Tumor/Lung | 0.67 ± 0.23 |  | 0.89 ± 0.06 | 2.74 ± 1.28 | 3.55 ± 0.64 | 5.70 ± 0.61 |
| Tumor/Liver | 0.48 ± 0.30 |  | 1.43 ± 0.05 | 3.27 ± 0.77 | 3.79 ± 0.39 | 5.78 ± 1.36 |
| Tumor/Spleen | 0.50 ± 0.20 |  | 1.36 ± 0.02 | 2.82 ± 0.85 | 4.68 ± 0.18 | 6.54 ± 2.81 |
| Tumor/Kidney | 1.17 ± 0.56 |  | 1.49 ± 0.30 | 3.54 ± 0.44 | 5.16 ± 0.13 | 7.71 ± 0.28 |
| Tumor/Muscle | 16.61 ± 7.61 |  | 12.54 ± 1.35 | 24.90 ± 2.74 | 36.93 ± 1.47 | 48.47 ± 5.39 |
| Tumor/Bone | 1.90 ± 1.03 |  | 4.64 ± 0.92 | 7.86 ± 3.86 | 21.17 ± 5.14 | 14.98 ± 5.44 |
