# Supplementary material for: Measuring the Pharmacodynamic Effects of a Novel Hsp90 Inhibitor on HER2/neu Expression in Mice Using 89Zr-DFO-Trastuzumab
Source: PLoS One. 2010 Jan 25;5(1):e8859. doi: 10.1371/journal.pone.0008859 (PMC2810330; doi:10.1371/journal.pone.0008859)
Supplement: Table S2 — A comparison of the difference between mean BT-474 tumor uptake values observed in the biodistribution studies in control (vehicle-treated) and PU-H71 treated mice. (0.04 MB DOC) [file pone.0008859.s008.doc]

| **Time / h** | Difference in mean  %ID/g values  (Control – PU-H71) | Tumor %ID/g ratio  (Control:PU-H71)*^a^* | *P*-value*^b^* |
| --- | --- | --- | --- |
| 12 | 9.25 | 1.64 | 0.100 |
| 24 | 34.93 | 2.17 | 0.0001 |
| 48 | 30.29 | 1.73 | 0.026 |
| 72 | 11.53 | 1.16 | 0.244 |

*^a^* Calculated from the mean tumor %ID/g values for control and PU-H71 treated animals. *^b^* *P*-value calculated between the control and PU-H71 treated %ID/g values at the specified time point.
