# Supplementary material for: Measuring the Pharmacodynamic Effects of a Novel Hsp90 Inhibitor on HER2/neu Expression in Mice Using 89Zr-DFO-Trastuzumab
Source: PLoS One. 2010 Jan 25;5(1):e8859. doi: 10.1371/journal.pone.0008859 (PMC2810330; doi:10.1371/journal.pone.0008859)
Supplement: Table S3 — Tumor-to-muscle (T/M) ratios have been calculated from volume-of-interest (VOI) analysis of immunoPET images recorded in dual tumor-bearing (BT-474 and MDA-MB-468) female, athymic nu/nu mice between 1–120 h post-i.v. administration of 89Zr-DFO-trastuzumab. The data presented are ratios of mean and maximum (%ID/g) values. Errors associated with VOI activity measurements are large and are strongly dependent on the number and definition of each ROI used in the determination of the VOI. As a consequence of the large errors associated with VOI analysis, and the further exaggeration that ensues from the calculation of ratios, errors associated with the calculated ratios are large, difficult to define and have been omitted to avoid misrepresentation of the data. (0.05 MB DOC) [file pone.0008859.s009.doc]

|  | Control (vehicle-treated) mice  (*n* = 3) | |  | | PU-H71 treated mice  (*n* = 3) | |
| --- | --- | --- | --- | --- | --- | --- |
| **Time / h** | BT-474 | MDA-MB-468 | | BT-474 | | MDA-MB-468 |
| *Mean T/M ratios* | |  | |  | |  |
| 1 | 1.55 | 1.52 | | 1.38 | | 2.72 |
| 5 | 7.87 | 2.26 | | 1.30 | | 1.16 |
| 7 | 7.88 | 2.20 | | 1.89 | | 1.94 |
| 24 | 7.63 | 2.82 | | 2.57 | | 3.00 |
| 48 | 7.85 | 2.85 | | 2.56 | | 2.94 |
| 72 | 6.71 | 3.17 | | 2.39 | | 3.10 |
| 120 | 6.77 | 5.53 | | 2.26 | | 2.67 |
|  |  |  | |  | |  |
| *Maximum T/M ratios* | |  | |  | |  |
| 1 | 3.57 | 3.28 | | 2.99 | | 2.34 |
| 5 | 11.90 | 4.15 | | 3.49 | | 2.65 |
| 7 | 12.38 | 3.82 | | 3.71 | | 3.37 |
| 24 | 13.43 | 4.88 | | 5.44 | | 5.21 |
| 48 | 16.09 | 4.69 | | 5.68 | | 4.76 |
| 72 | 16.24 | 5.09 | | 6.18 | | 4.94 |
| 120 | 18.30 | 7.55 | | 5.29 | | 4.16 |
