# Supplementary material for: Measuring the Pharmacodynamic Effects of a Novel Hsp90 Inhibitor on HER2/neu Expression in Mice Using 89Zr-DFO-Trastuzumab
Source: PLoS One. 2010 Jan 25;5(1):e8859. doi: 10.1371/journal.pone.0008859 (PMC2810330; doi:10.1371/journal.pone.0008859)
Supplement: Table S4 — Summary of the calculated effective and biological half-lives (0.04 MB DOC) [file pone.0008859.s010.doc]

| Parameter | BT-474 mice | MDA-MB-468 mice |
| --- | --- | --- |
| λ_eff_ / h^-1^ | 0.01388 ± 0.00182 | 0.01172 ± 0.00169 |
| t_1/2.eff_ / h | 50.5 ± 6.4 | 60.1 ± 8.4 |
| t_1/2.physical_ / h | 78.41 | 78.41 |
| t_1/2.biological_ / h | 150.5 ± 49.5 | 336.2 ± 184.5 |
